# Supplementary material for: Individualized phenotyping of functional amyotrophic lateral sclerosis pathology in sensorimotor cortex
Source: Brain Commun. 2026 Apr 9;8(2):fcag127. doi: 10.1093/braincomms/fcag127 (PMC13122844; doi:10.1093/braincomms/fcag127)
Supplement: fcag127_Supplementary_Data [file fcag127_supplementary_data.pdf]

Supplementary Material

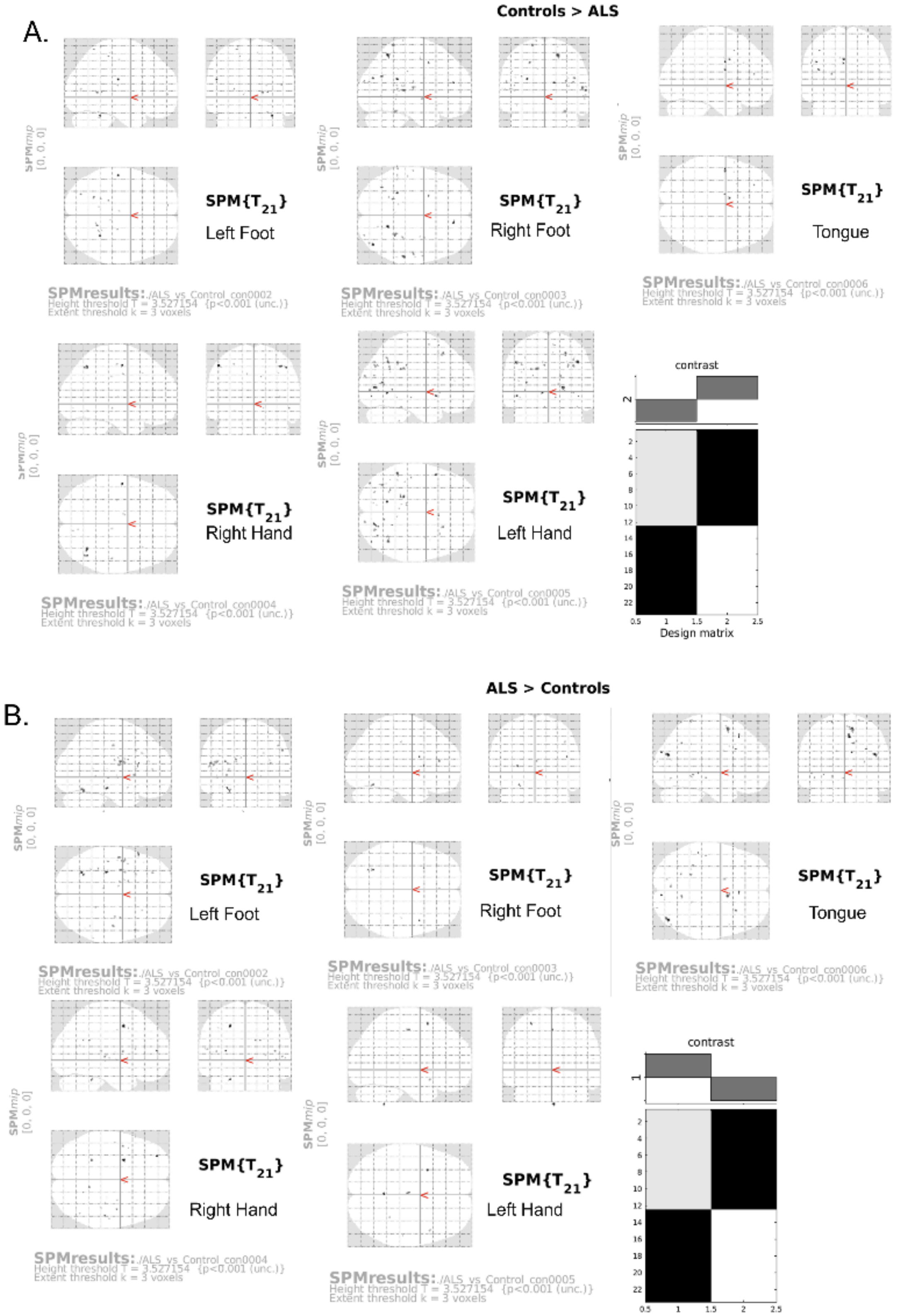

Supplementary Figure 1: Group-level GLM results showing voxel-wise T-contrasts between ALS and control participants. Statistical parametric maps (SPM{T}) show results from second-level two-sample t-tests for each region-specific contrast derived from first-level GLM analysis. Contrasts correspond to activations in specific sensorimotor regions: left foot, right foot, left hand, right hand, and tongue. The contrast (A) *Control > ALS* and (B) *ALS > Control* is thresholded at $p < 0.001$ (uncorrected, N= 12), with a cluster extent threshold of $k = 3$ voxels. No clusters survived multiple comparison correction (FWE), but uncorrected maps highlight distributed sub-threshold group differences. The design matrix used for this second-level model is shown at the bottom right side of panels (A) and (B).

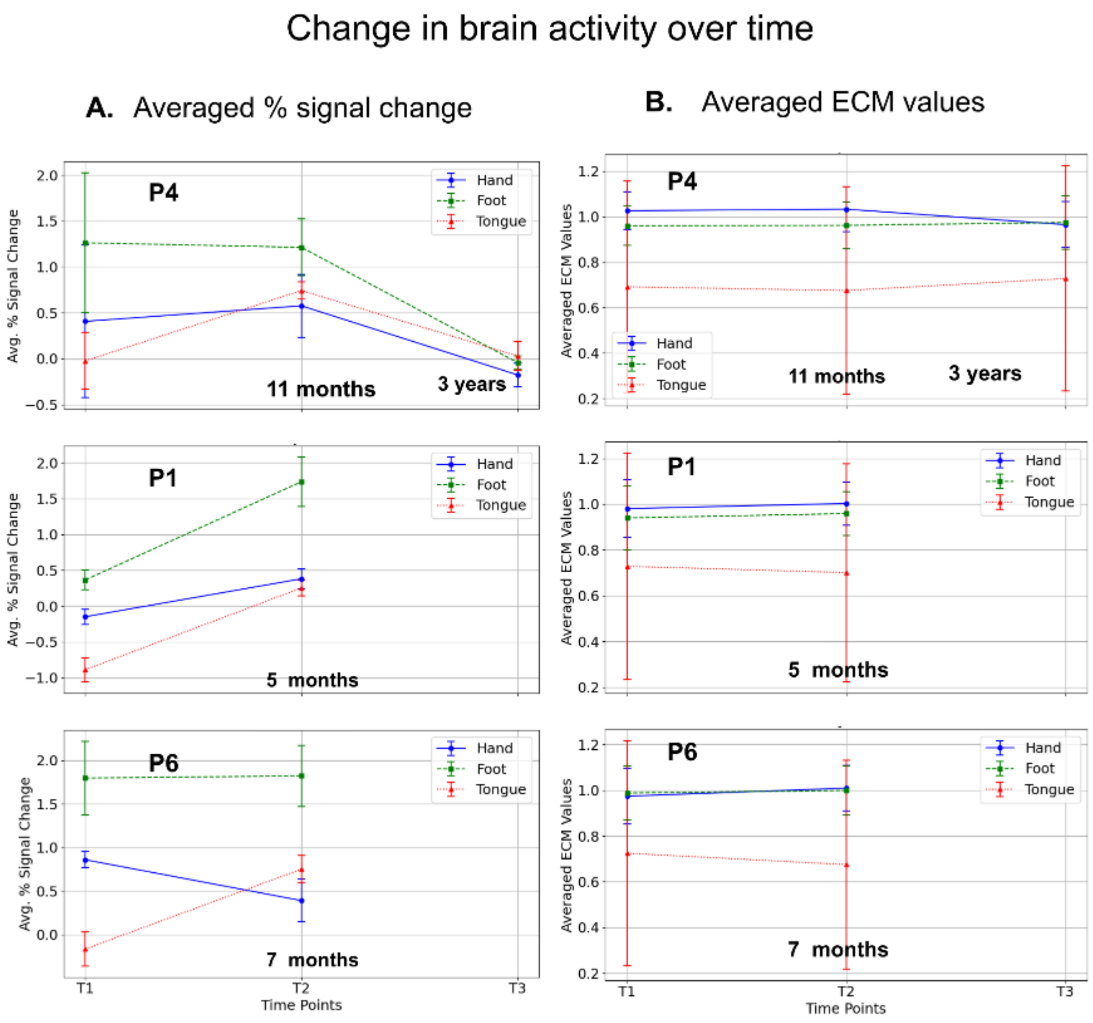

Clinical assessment scores over time

Patients	T1	T2	T3
P4	ALSFRS-R Total: **37** (bulbar: 12, fine : 6, gross : 7, breathing: 12), KS: **2**	ALSFRS-R Total: **36** (bulbar: 12, fine : 6, gross : 7, breathing: 11), KS: **2**	ALSFRS-R Total: **41** (bulbar: 11, fine : 10, gross : 9, breathing: 11), KS: **2**
P1	-	ALSFRS-R Total: **44** (bulbar: 12, fine : 9, gross : 11, breathing: 12) KS:1	-
P6	ALSFRS-R Total: **41** (bulbar: 9, fine : 9, gross : 11, breathing: 12) KS: **2**	ALSFRS-R Total: **38** (bulbar: 9, fine : 7, gross : 10, breathing: 12) KS: **3**	

Supplementary Figure 2: Changes in % signal change and functional connectivity over time: (A) Averaged % signal change and **(B)** averaged Eigenvector Centrality Mapping (ECM) Values: Line graphs illustrate averaged % signal change and ECM values in brain activity for three subjects across different time points: +11 months and +3 years after the first san for P4, +5 months after the first scan for P1, +7 months after the first scan for P6. Data is extracted from the hand (blue), foot (red), and tongue (green)

regions in MI, highlighting the variations in functional activation and connectivity over time. The table at the bottom row shows the behavioral ALSFRS-R scores measures over time, and the King Stage (KS) for the patients. Note: P1 was not recorded at the first time point

Supplementary Table 1 Percent signal change and connectivity changes in ALS patients over time

Patient	Region	Time Point	Percent Signal Change (mean ± SEM)	ECM Values (mean ± SEM)
P4	Hand	Baseline	0.41 ± 0.83	1.03 ± 0.08
		11 months	0.58 ± 0.34	1.03 ± 0.10
		3 years	0.18 ± 0.13	0.97 ± 0.12
	Foot	Baseline	1.26 ± 0.76	0.96 ± 0.09
		11 months	1.21 ± 0.31	0.96 ± 0.10
		3 years	0.04 ± 0.08	0.96 ± 0.10
	Bulbar	Baseline	0.02 ± 0.31	0.69 ± 0.46
		11 months	0.74 ± 0.09	0.68 ± 0.46
		3 years	0.03 ± 0.16	0.73 ± 0.50
P1	Hand	Baseline	0.15 ± 0.11	0.98 ± 0.13
		5 months	0.38 ± 0.14	1.00 ± 0.09
	Foot	Baseline	0.37 ± 0.14	0.94 ± 0.14
		5 months	1.74 ± 0.34	0.96 ± 0.09
	Bulbar	Baseline	-0.89 ± 0.17	0.73 ± 0.49
		5 months	0.25 ± 0.11	0.70 ± 0.48
P6	Hand	Baseline	0.86 ± 0.10	0.97 ± 0.12
		7 months	0.39 ± 0.24	1.01 ± 0.10
	Foot	Baseline	1.80 ± 0.42	0.99 ± 0.12
		7 months	1.82 ± 0.35	1.00 ± 0.11
	Bulbar	Baseline	0.16 ± 0.20	0.72 ± 0.49
		7 months	0.75 ± 0.16	0.67 ± 0.46

.

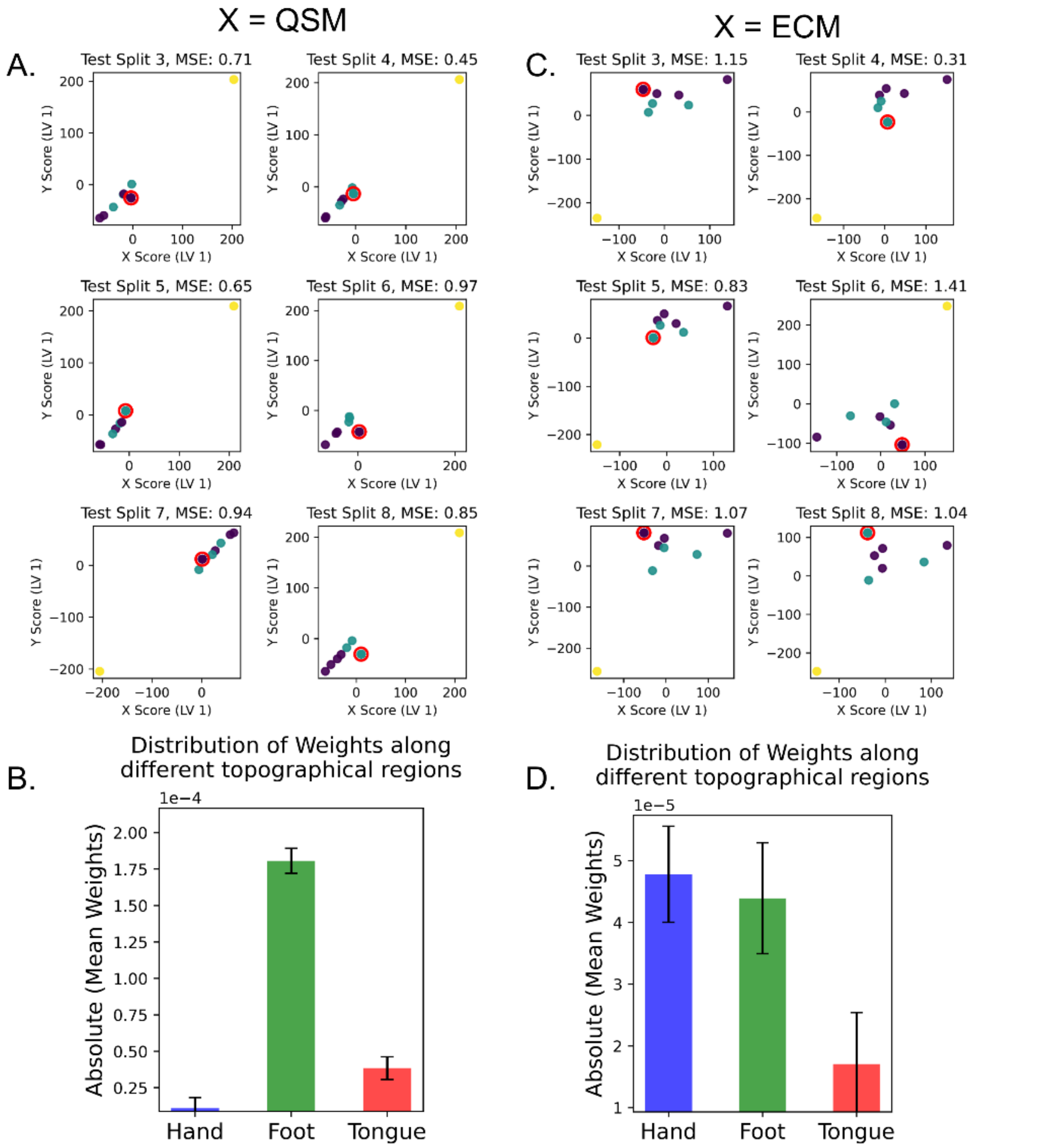

Supplementary Figure 3: Comparison of latent space projections and topographic weight distributions from QSM and ECM data. (A) PLSR-based cross-validation splits using QSM (left) and ECM (right **C**) as input features, showing latent variable (LV1) projections with subject-level test set MSEs indicated. Red-circled points represent held-out subjects. **(B)** Average absolute region weights (hand, foot, tongue) from QSM-derived LV1s across subjects (N = 8 ALS patients). **(D)** Average absolute region weights from ECM-derived LV1s (N = 8 ALS patients). These distributions indicate differing topographic emphasis between structural and functional modalities.

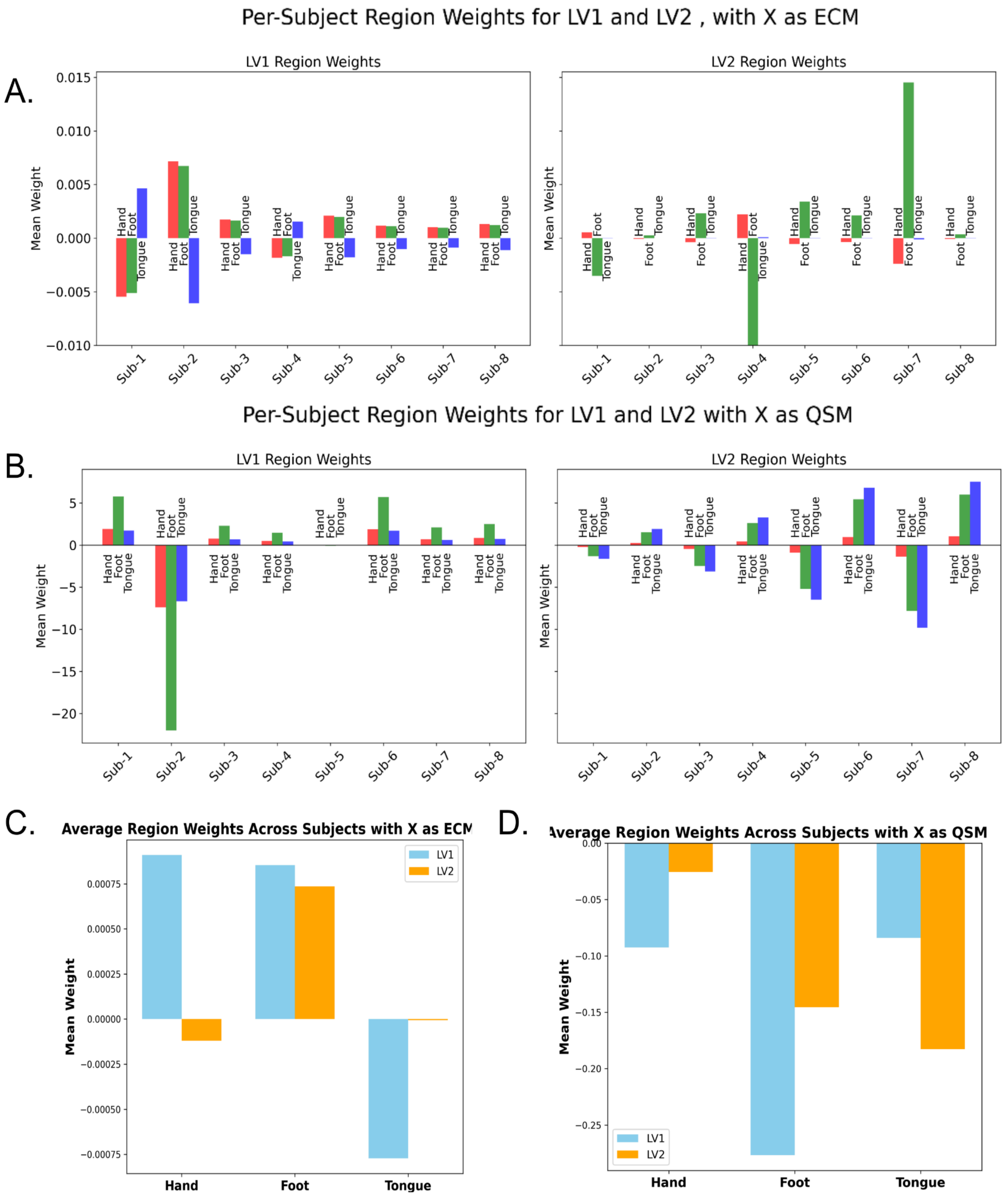

Supplementary Figure 4: Subject-wise and group-level region weights across latent dimensions derived from QSM and ECM data. (A) LV1 and LV2 region weights per subject using ECM data as input (X = ECM). **(B)** LV1 and LV2 region weights per subject using QSM data as input (X = QSM). **(D–E)** Mean region weights across subjects, grouped by LV1 and LV2, for ECM (panel D) and QSM (panel E), respectively. The two modalities emphasize different sensorimotor topographies, suggesting they capture distinct disease-relevant features.

S5. Feature Space Representation and Classification Performance

To further examine the classification performance of ALS and control participants, we analyzed fMRI data from the later-affected body regions and their matched control data. We aimed to explore the structure of the feature space that supported successful group separation. Scatter plots from Principal Component Analysis (PCA) and t-distributed Stochastic Neighbor Embedding (t-SNE) illustrate distinct group-level separation, particularly in the shared response space.

We also evaluated the classifier's performance using leave-one-out cross-validation on the SRM-derived features. The resulting confusion matrix (**Supplementary Figure 5 (C))** yielded the following totals across folds: True Positives = 112, True Negatives = 121, False Negatives = 9, and False Positives = 0, corresponding to an overall classification accuracy of approximately 96%.

Supplementary Figure 5 (A–B) illustrates the impact of SRM preprocessing on separability. SRM-aligned data (Panel A) yielded clearer clustering of ALS and control participants in low-dimensional representations, while raw time series data (Panel B) showed greater overlap. This improved separation likely contributes to the robust classification performance achieved using SRM-derived features.

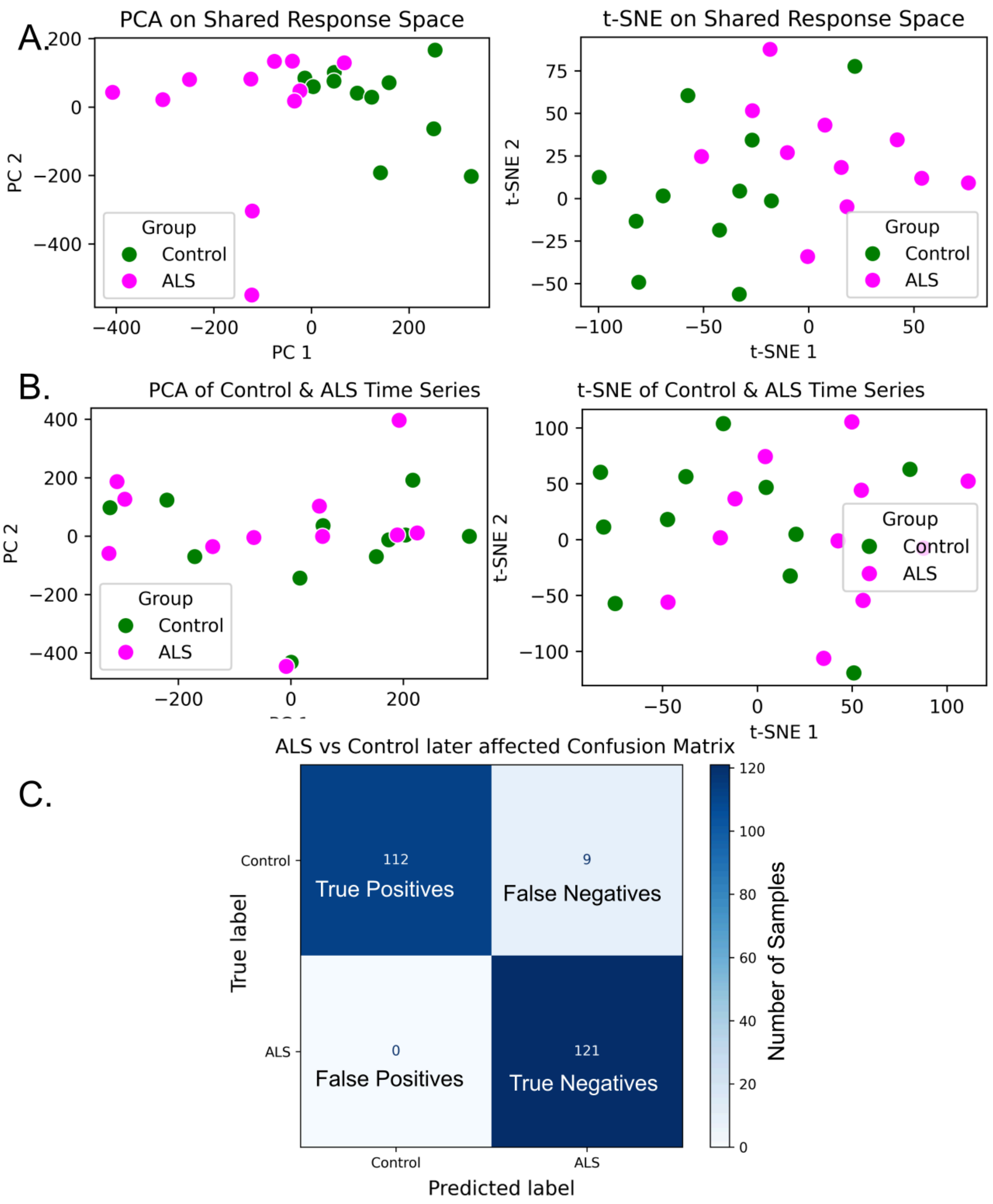

Supplementary Figure 5: Classification performance and latent space separability between ALS and control groups. **(A)** Dimensionality reduction of SRM-aligned data using Principal Component Analysis (PCA) (left) and t-distributed Stochastic Neighbor Embedding (t-SNE) (right) illustrates group-wise separation in the shared functional space. ALS and control participants form partially distinct clusters (*green* for Control and *magenta* for ALS). **(B)** Dimensionality reduction of unaligned raw time series (rSRM input) using PCA (left) and t-SNE (right). These results highlight the utility of SRM in enhancing group-level differentiation in functional patterns. **(C)** Confusion matrix showing classification results of ALS vs. control participants using SRM-projected features and a linear SVM model. The rows show the true group (Control top, ALS bottom) and columns show the predicted group (Control left, ALS right), with cell counts indicating correctly classified (diagonal) and misclassified (off-diagonal) samples.
